# Changes in the calorie and nutrient content of purchased fast food meals after calorie menu labeling: A natural experiment

**DOI:** 10.1371/journal.pmed.1003714

**Published:** 2021-07-12

**Authors:** Joshua Petimar, Fang Zhang, Eric B. Rimm, Denise Simon, Lauren P. Cleveland, Steven L. Gortmaker, Sara N. Bleich, Michele Polacsek, Christina A. Roberto, Jason P. Block

**Affiliations:** 1 Division of Chronic Disease Research Across the Lifecourse, Department of Population Medicine, Harvard Pilgrim Health Care Institute & Harvard Medical School, Boston, Massachusetts, United States of America; 2 Department of Epidemiology, Harvard T.H. Chan School of Public Health, Boston, Massachusetts, United States of America; 3 Division of Health Policy and Insurance Research, Department of Population Medicine, Harvard Pilgrim Health Care Institute & Harvard Medical School, Boston, Massachusetts, United States of America; 4 Department of Nutrition, Harvard T.H. Chan School of Public Health, Boston, Massachusetts, United States of America; 5 Department of Social and Behavioral Sciences, Harvard T.H. Chan School of Public Health, Boston, Massachusetts, United States of America; 6 Department of Health Policy and Management, Harvard T.H. Chan School of Public Health, Boston, Massachusetts, United States of America; 7 Westbrook College of Health Professions, University of New England, Portland, Maine, United States of America; 8 Department of Medical Ethics and Health Policy, Perelman School of Medicine, University of Pennsylvania, Philadelphia, Pennsylvania, United States of America; University of Cambridge, UNITED KINGDOM

## Abstract

**Background:**

Calorie menu labeling is a policy that requires food establishments to post the calories on menu offerings to encourage healthy food choice. Calorie labeling has been implemented in the United States since May 2018 per the Affordable Care Act, but to the best of our knowledge, no studies have evaluated the relationship between calorie labeling and meal purchases since nationwide implementation of this policy. Our objective was to investigate the relationship between calorie labeling and the calorie and nutrient content of purchased meals after a fast food franchise began labeling in April 2017, prior to the required nationwide implementation, and after nationwide implementation of labeling in May 2018, when all large US chain restaurants were required to label their menus.

**Methods and findings:**

We obtained weekly aggregated sales data from 104 restaurants that are part of a fast food franchise for 3 national chains in 3 US states: Louisiana, Mississippi, and Texas. The franchise provided all sales data from April 2015 until April 2019. The franchise labeled menus in April 2017, 1 year prior to the required nationwide implementation date of May 2018 set by the US Food and Drug Administration. We obtained nutrition information for items sold (calories, fat, carbohydrates, protein, saturated fat, sugar, dietary fiber, and sodium) from Menustat, a publicly available database with nutrition information for items offered at the top revenue-generating US restaurant chains. We used an interrupted time series to find level and trend changes in mean weekly calorie and nutrient content per transaction after franchise and nationwide labeling. The analytic sample represented 331,776,445 items purchased across 67,112,342 transactions. Franchise labeling was associated with a level change of −54 calories/transaction (95% confidence interval [CI]: −67, −42, *p* < 0.0001) and a subsequent 3.3 calories/transaction increase per 4-week period (95% CI: 2.5, 4.1, *p* < 0.0001). Nationwide implementation was associated with a level decrease of −82 calories/transaction (95% CI: −88, −76, *p* < 0.0001) and a subsequent −2.1 calories/transaction decrease per 4-week period (95% CI: −2.9, −1.3, *p* < 0.0001). At the end of the study, the model-based predicted mean calories/transaction was 4.7% lower (change = −73 calories/transaction, 95% CI: −81, −65), and nutrients/transaction ranged from 1.8% lower (saturated fat) to 7.0% lower (sugar) than what we would expect had labeling not been implemented. The main limitations were potential residual time-varying confounding and lack of individual-level transaction data.

**Conclusions:**

In this study, we observed that calorie labeling was associated with small decreases in mean calorie and nutrient content of fast food meals 2 years after franchise labeling and nearly 1 year after implementation of labeling nationwide. These changes imply that calorie labeling was associated with small improvements in purchased meal quality in US chain restaurants.

## Abstract 2

In an interrupted time series analysis, Joshua Petimar and colleagues investigate the relationship between the implementation of calorie content labelling on fast food restaurant menus and changes in calorie and nutrition content of meal purchases from a restaurant franchise in the US.

## Author summary

**Why was this study done?:**

- The 2010 Affordable Care Act required that all chain food establishments with ≥20 locations in the United States label their menus with calorie content of prepared foods to encourage healthier eating.
- There is little evidence on how the nutritional quality of purchased meals changed after nationwide implementation of this policy.

**What did the researchers do and find?:**

- We analyzed sales data from a franchise of 104 fast food restaurants (representing 3 large nationwide chains in the US) to determine how nutrient purchases changed after voluntary franchise labeling in April 2017 and nationwide implementation of labeling in May 2018.
- At the end of the study, nearly 1 year after nationwide implementation of calorie labeling, the average calorie content of meals was 4.7% lower than what would be expected had labeling not occurred.
- Other nutrients ranged from 1.8% lower (saturated fat) to 7.0% lower (sugar) at the end of the study, but the nutrient density of purchases still generally exceeded US dietary guidelines.

**What do these findings mean?:**

- Calorie menu labeling is a promising strategy for improving purchased meal quality in fast food restaurants, but the effect is small.
- Future food retail interventions may be necessary to make further improvements in meal quality in restaurants.

## Introduction

As global eating patterns increasingly shift toward increased consumption of foods prepared away from home [1–3], various strategies are needed to promote healthy food choices in retail environments. To increase transparency and encourage healthy eating, many countries have introduced or implemented nutritional labeling policies such as menu labeling, front-of-package nutrition disclosures, warning labels, and others [4]. Calorie menu labeling, which requires food outlets to post the calorie content of menu offerings, is one such strategy that has been implemented in several countries, including the United States, South Korea, and Saudi Arabia [5]. As other countries consider passage or implementation of similar menu labeling policies, more real-world evidence is needed regarding the effect they have on customer meal choices.

In May 2018, the US Food and Drug Administration required implementation of calorie menu labeling in food establishments with ≥20 locations, which was mandated by the 2010 Affordable Care Act (ACA) [6]. Evidence from rigorous quasi-experimental evaluations of this policy is mixed; smaller studies have generally found no change in the calorie or nutrient content of consumer purchases after labeling [7–13], but studies examining large databases of retail transactions have detected small decreases in meals’ calorie content [14,15]. However, to our knowledge, no studies have examined these associations since nationwide implementation of this policy. Nationwide implementation may have led to improvements in customer meal purchases because of increased attention surrounding the policy and because customers were exposed to labeling at nearly all large chain restaurants [16]. Both are mechanisms that may have enhanced awareness of the policy and encouraged healthier purchasing. Restaurants also may have reformulated their menus to reduce the calorie content of offerings after labeling, which could reduce customer calorie purchases without requiring changes to their behavior [5]. Evidence of reformulation has not been observed in Canada [17] or Australia [18] after implementation of regional labeling laws, but no studies to our knowledge have investigated reformulation after nationwide implementation of menu labeling. Evidence on post-implementation effects of US calorie labeling is thus important to gain a broader understanding of the effect of this policy and to offer guidance to policymakers in countries where it is being considered.

Using sales data from a large fast food franchise in the southern US that voluntarily implemented calorie labeling in April 2017, we previously found that the calorie content of purchased meals declined by 4% after labeling, with an increasing trend that attenuated this association over the year that followed [14]. This study ended 1 month before calorie labeling was implemented nationwide. We have obtained an additional year of sales data from the franchise, which allows us to now report changes in calorie and nutrient content of meals purchased 2 years after franchise calorie labeling (April 2017 to April 2019), including an examination of whether purchases changed specifically after nationwide implementation of labeling (May 2018 to April 2019).

## Methods

We leveraged a natural experiment to determine how the nutrient content of purchased meals in fast food restaurants changed after franchise calorie menu labeling and nationwide implementation of calorie labeling. We used data from a 2-year pre-labeling period to predict what meals’ nutrient content would have been in the post-labeling period had labeling not happened; we compared these to the observed nutrient content to estimate the effect of labeling.

### Data source

We obtained sales data from a fast food franchise with 143 restaurants from 3 national chains (all under 1 large national company) with locations in Louisiana, Mississippi, and Texas [14]. In 2017 (midway through follow-up), these 3 chains were all among the top 100 largest restaurant chains and collectively had over 11,000 US locations and nearly $15 billion in sales [19]. For each of the 143 restaurants, the franchise provided weekly sales data from April 2015 until April 2019, including the total units purchased of each menu offering and the total number of transactions. The franchise labeled all menus during the week of April 6, 2017. After several delays [20], the US Food and Drug Administration required nationwide labeling of all chain food establishments beginning the week of May 7, 2018.

We included 104 restaurants with sales data in the pre-labeling period (April 2015 to April 2017), franchise labeling period (April 2017 to May 2018), and nationwide labeling period (May 2018 to April 2019); these are the same 104 restaurants included in our previous study [14]. We excluded 176 restaurant-weeks (0.8% of all observations) with missing sales or transaction data. These missing observations occurred across 19 different restaurants without any significant temporal or geographic pattern and may have been due to temporary store closures (e.g., due to construction). We additionally excluded sales data for 40 restaurants (0.2% observations) in Texas during the week of Hurricane Harvey because of very few transactions, as well as 19 observations (<0.1%) where the total number of items purchased or the mean calories/transaction were outliers. For example, some stores documented weeks with approximately 1% of their normal sales volume, indicating the store may have been closed and should have been counted as missing. The final dataset included 20,563 weeks of transaction data. We submitted an analysis plan (located at www.aspredicted.org/4xx8v.pdf and in **S1 Appendix**) before conducting analysis in our previously published study [14]. While the study dataset and outcomes assessed were more comprehensive, the methods we used in the present study were very similar. As a result, we did not submit a new analysis plan. We have provided information about changes we made to the prepublished analytic approach in **S2 Appendix**.

### Outcomes

The primary outcome was mean calories/transaction. This was calculated as the total calories purchased in a restaurant in a week divided by the total number of transactions purchased in that restaurant in that week. Secondary outcomes were mean nutrients/transaction, including total fat (g), total carbohydrates (g), total protein (g), saturated fat (g), sugar (g), dietary fiber (g), and sodium (mg). These were calculated similarly to how we calculated mean calories/transaction. We calculated these by identifying each menu item using a unique transaction code and item description. We then matched each item to its corresponding entry in Menustat, a publicly available database created by the New York City Department of Health and Mental Hygiene (DOHMH) with nutrition information for items offered at the top revenue-generating US restaurant chains, including the chains in this study [21]. New Menustat databases were released every year except 2019. Using the same methods and guidance from DOHMH [22] (see **S2 Appendix**), we obtained information for foods sold in 2019 by creating our own database with nutrition data for items at the same top restaurant chains. We matched 94.3% of purchased items using this approach. If an item was not listed in Menustat for any year, we used a different year in Menustat (3.0% of items) or nutrition information from the restaurant website if available (2.6%). We deleted the remaining unmatched purchased items (0.2%).

To calculate the primary outcome of mean calories/transaction, we multiplied the total number of each item purchased by the item’s calorie content for each restaurant in a given week, summed over all items, and divided by the total number of transactions in that restaurant in that week. We repeated this for each nutrient of interest and calculated the mean nutrient/transaction (in g/transaction or mg/transaction).

Starting in early 2017, the franchise changed methods of recording combo meals such that both the combo and its components were recorded (i.e., duplicating purchases; before 2017 only the combo was recorded). In the period after this change took place, we were unable to distinguish between items purchased as part of combos and items purchased a la carte. To correct this, we deleted all combos from restaurants’ sales data after this change took place and “unbundled” combos from the period before this change (16% of total purchased items) to match the format of the following period. To unbundle the combos, we identified all items that came with the combo using information from restaurant websites (if available) or from past advertisements or news articles. If more than 1 option was available for any given combo component (e.g., choice of chicken or beef), we chose the default option or the option that did not require additional charges. Identification of combo components and the appropriate default option was done by 1 member of the research team, and 2 other members reviewed these decisions. Any disagreements were resolved by consensus. In previous work, we found that our results were robust to changing which option was selected for a combo component when more than one was available [14].

### Other measures

We measured the socioeconomic status of the neighborhood in which each restaurant was located using data on median household income in the restaurant’s census tract. In the US, a census tract is a small geographic unit (typically having a population size between 1,200 and 8,000 people) for which population-level demographic data are available [23]. We linked census tract–level data from the most recent 5-year American Community Survey (ACS) (2013 to 2017) to geocoded restaurant census tracts [24].

### Statistical analysis

We used interrupted time series (ITS) with segmented regression [25] to estimate the change in mean calories/transaction and nutrients/transaction after franchise labeling and nationwide labeling. This approach used the 2-year trend in the pre-intervention data to predict what the outcome would have been in the post-intervention periods in the absence of labeling (i.e., the counterfactual) [25,26]. Our model included indicator variables for season and the winter holiday period, which improved the model fit for the pre-labeling data [14]. The model included terms for franchise labeling (β_franchise_) and week-after-franchise labeling (β_weekfranchise_), which estimated the change in the level (i.e., intercept) and trend in the outcome of interest, respectively, after franchise labeling. The model also included terms for nationwide labeling (β_nation_) and week-after-nationwide labeling (β_weeknation_), which estimated the level and trend change, respectively, after nationwide labeling compared to what we would expect had nationwide labeling not been implemented. The full model is provided in S2 Appendix.

We excluded the week of labeling implementation and the 2 weeks before and after (for both franchise and nationwide labeling) to account for potential variation in rollout. We used a linear mixed model with random intercepts at baseline and at each labeling period and robust standard errors. This accounted for possible correlation between measures of the same restaurants over time, clustering of purchases within restaurants, and different intercepts and level changes between restaurants. Because some estimates were very small using a 1-week increment, we multiplied parameter estimates for trend changes by 4 to calculate trends per 4-week period. To evaluate the effect of labeling at the end of the study period, we estimated actual and counterfactual values of outcomes in the last week using model coefficients, calculated the difference between these values, and obtained 95% confidence intervals (CIs) for the difference from 1,000 bootstrapped samples.

We conducted sensitivity analyses to test the robustness of our findings for calories/transaction. First, we included 1 year of pre-labeling data, which assumes a shorter and more proximal time period to capture the baseline trend. Second, we included only restaurants with complete data for all weeks of the study (*n =* 31 because many locations briefly opened or closed throughout the study period). Third, we calculated the mean calories/transaction over 4-week periods instead of 1-week periods to smooth out potentially large week-to-week fluctuations in calories/transaction that could have reduced model accuracy. Fourth, instead of including indicator variables for season, we included sine and cosine terms [27], which might better capture yearly seasonal trends in calories/transaction. Last, our original hypothesis was that nationwide labeling could affect customers’ purchases by newly exposing them to labeling at other restaurants, but many chain restaurants started labeling their menus before the nationwide implementation date. For all outcomes, we ran a model assuming level and trend changes only at the time of franchise labeling, which could be more appropriate if nationwide labeling rollout was more relevant than the nationwide implementation date.

We conducted stratified analyses by median income of restaurant census tracts (above/below the median of $50,329/household) because we previously found a stronger trend increase in calories/transaction in lower-income census tracts after franchise labeling [14]. We conducted exploratory analyses to generate hypotheses about mechanisms driving associations of calorie labeling with purchases. First, we explored the role of potential product reformulation in restaurants, which could reduce calorie content of meals without any behavioral change. We identified the top 100 high-selling items that were sold both before and after labeling in our sample. We ran separate linear regressions with calories and nutrients as the dependent variables, indicators for year as dependent variables, and item-level fixed effects to calculate the mean changes in 2018 and 2019 compared to the pre-labeling period (2015 to 2017). We also examined items that were not sold in 2015 (the first year of the study) but were sold sometime after. We determined whether the item was newly introduced before labeling (in 2016 to 2017) or after labeling and ran a regression to determine whether the mean nutrient content was lower for items newly introduced after labeling compared to those newly introduced before labeling. Second, we examined the distribution of calories and nutrients among menu options by year to assess the role of changes in menu offerings. Lastly, we explored whether consumers purchased overall lower-calorie items or purchased fewer items per transaction after labeling by rerunning our ITS models but using calories/item and items/transaction as the dependent variable.

We used SAS version 9.4 (Cary, North Carolina) and calculated 2-sided 95% CIs for all statistical tests. This study was reviewed and approved by the institutional review board of Harvard Pilgrim Health Care. This study is reported as per the Strengthening the Reporting of Observational Studies in Epidemiology (STROBE) guideline (S1 Checklist).

## Results

Our final sample included 59 restaurants in Louisiana, 41 in Texas, and 4 in Mississippi. On average, each restaurant had sales data available for 198 weeks. Our analytic dataset represented 332 million items purchased and 67 million transactions over the study period (**Table 1**). In the pre-labeling period, there were about 4.9 items purchased per transaction, and transactions had a mean (SD) of 1,486 calories (152), 63.5 g fat (7.6), 182.5 g carbohydrate (16.8), 50.2 g protein (6.1), 20.6 g saturated fat (2.5), 67.0 g sugar (7.0), 16.5 g fiber (1.7), and 2,841 mg sodium (378).

**Table 1:** Transactions, item purchases, and mean (SD) calorie and nutrient content of purchased meals in restaurants overall and by calorie labeling implementation period.

		Pre-labeling	Post-labeling (franchise:	Post-labeling (nationwide:
Characteristic	Total	(April 2015 to April 2017)	April 2017 to May 2018)	May 2018 to April 2019)
Transactions	67,112,342	31,006,881	18,759,891	17,345,570
Items purchased	331,776,445	152,724,842	92,602,285	86,449,318
**Mean (SD) nutrient content per transaction**				
Calories	1,480 (140)	1,486 (152)	1,483 (136)	1,465 (121)
Fat (g)	62.7 (6.7)	63.5 (7.6)	62.6 (6.2)	61.5 (5.4)
Carbohydrates (g)	183.1 (16.1)	182.5 (16.8)	183.0 (15.9)	184.2 (14.9)
Protein (g)	49.7 (5.6)	50.2 (6.1)	50.1 (5.4)	48.4 (4.6)
Saturated fat (g)	20.3 (2.2)	20.6 (2.5)	20.4 (2.1)	19.8 (1.8)
Sugar (g)	67.3 (6.7)	67.0 (7.0)	67.6 (6.5)	67.5 (6.5)
Fiber (g)	16.6 (1.7)	16.5 (1.7)	16.7 (1.7)	16.7 (1.6)
Sodium (mg)	2,814 (336)	2,841 (378)	2,822 (309)	2,761 (280)

SD, standard deviation.

**Fig 1:**
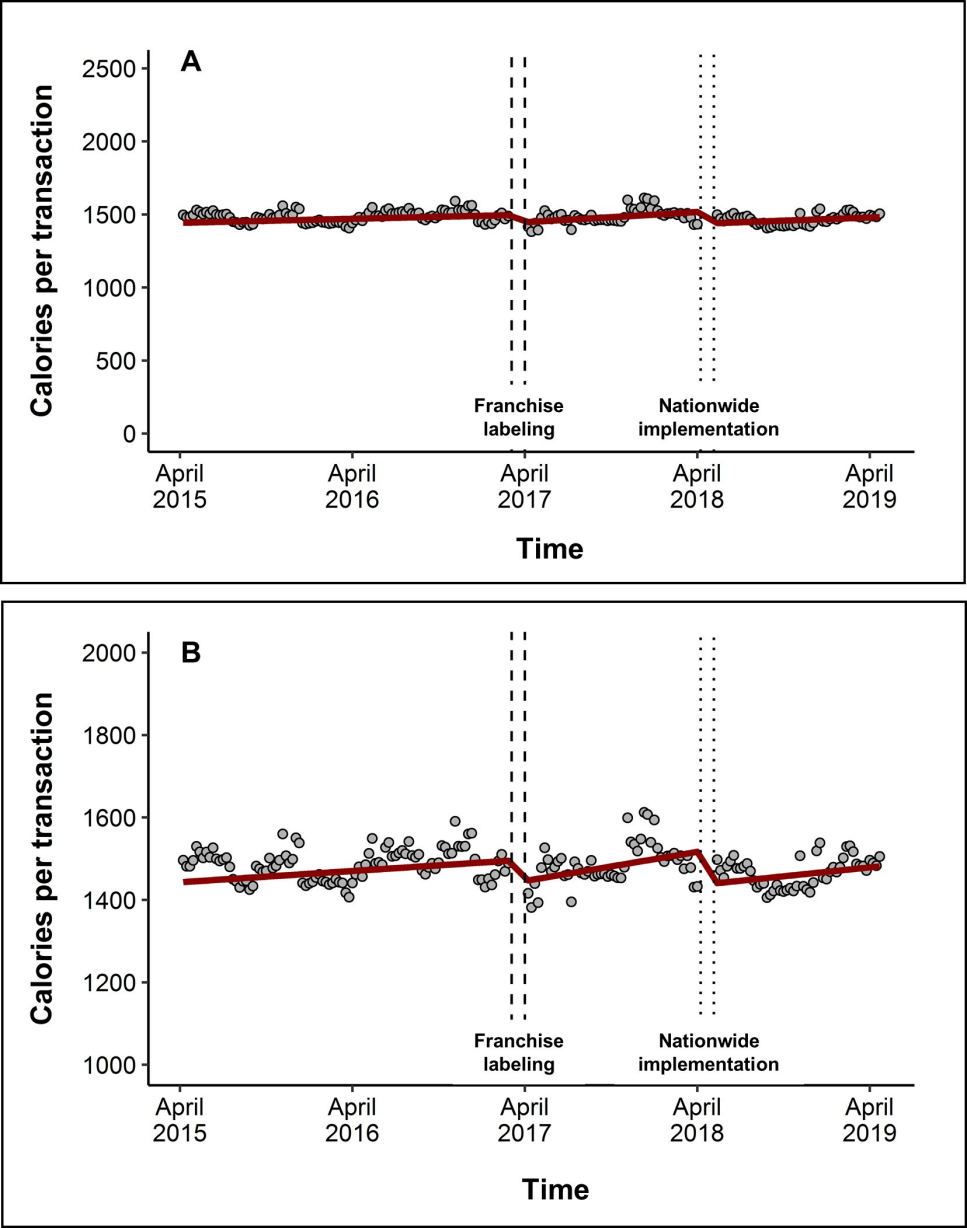
Level and trend changes in mean purchased calories per transaction after franchise labeling and implementation of labeling nationwide. (A) The graph shows mean calories/transaction across all restaurants (gray dots) and the predicted calories/transaction from the model (red line). The model is also adjusted for seasons and holidays, but these effects are not depicted here. The time surrounding franchise labeling (April 2017) is depicted as dashed vertical lines, and the time surrounding nationwide implementation (May 2018) is depicted as dotted lines. (B) Same as (A) but magnified to see level and trend changes more easily.

Franchise labeling in April 2017 was associated with a level decrease of −54 calories/transaction (95% CI: −67, −42, *p* < 0.0001) and a trend increase of 3.3 calories/transaction per 4-week period (95% CI: 2.5, 4.1, *p* < 0.0001) over the subsequent 13 months that was independent of the baseline trend. Nationwide implementation of labeling in May 2018 was associated with another level decrease of −82 calories/transaction (95% CI: −88, −76, *p* < 0.0001) and a trend decrease of −2.1 calories/transaction per 4-week period (95% CI: −2.9, −1.3, *p* < 0.0001) (**Fig 1, Table 2**). The overall change in trend after franchise implementation, inclusive of both the post-franchise and post-nationwide labeling periods, was still positive compared to the baseline trend (i.e., [β_weekfranchise_ = 3.3] + [β_weeknation_ = −2.1] = 1.2 calories/transaction per 4-week period [95% CI: 0.80, 1.63 from 1,000 bootstrapped samples]). In the last week of the study, the mean transaction contained 73 (95% CI: 65, 81) fewer calories than what would be expected had labeling not been implemented, a 4.7% decrease. We observed generally similar results in sensitivity analyses (**S1 Table**). However, when using only 1 year of pre-labeling data, the model predicted that by the end of the study, the mean transaction increased by 43 calories (95% CI: 22, 63).

**Table 2:** Interrupted time series for change in mean calories and mean nutrients purchased per transaction after franchise calorie labeling implementation (April 2017) and after nationwide calorie labeling implementation (May 2018).

	β (95% CI)^1^						
Nutrient	Baseline level	Baseline trend^2^	Franchise level change^3^	Franchise trend change^4^	Nationwide level change^5^	Nationwide trend change^6^	Estimated change at end of study^7^
**Calories per transaction**	1,443 (1,411, 1,474)	2.1 (1.5, 2.7)	−54 (−67, −42)	3.3 (2.5, 4.1)	−82 (−88, −76)	−2.1 (−2.9, −1.3)	−73 (−81, −65)
**Absolute nutrient content per transaction**							
Fat (g)	62.3 (60.7, 63.9)	0.0 (0.0, 0.1)	−1.2 (−2.0, −0.5)	0.1 (0.1, 0.2)	−2.5 (−2.8, −2.2)	−0.1 (−0.2, −0.1)	−1.9 (−2.3, −1.5)
Carbohydrates (g)	175.9 (172.6, 179.3)	0.4 (0.4, 0.5)	−10.1 (−11.3, −9.0)	0.5 (0.4, 0.5)	−9.7 (−10.4, −9.0)	−0.2 (−0.3, −0.1)	−9.9 (−10.8, −8.9)
Protein (g)	48.6 (47.3, 49.9)	0.0 (0.0, 0.1)	−0.1 (−0.6, 0.5)	0.0 (−0.1, 0.0)	−2.5 (−2.8, −2.3)	0.1 (0.1, 0.1)	−2.6 (−2.9, −2.3)
Saturated fat (g)	20.2 (19.7, 20.8)	0.0 (0.0, 0.0)	−0.4 (−0.7, −0.1)	0.1 (0.1, 0.1)	−1.5 (−1.6, −1.4)	0.0 (0.0, 0.0)	−0.4 (−0.5, −0.2)
Sugar (g)	65.0 (63.6, 66.5)	0.2 (0.1, 0.2)	−1.9 (−2.4, −1.4)	0.0 (−0.1, 0.0)	−1.1 (−1.4, −0.7)	−0.1 (−0.1, 0.0)	−5.1 (−5.5, −4.7)
Fiber (g)	15.7 (15.4, 16.0)	0.0 (0.0, 0.1)	−0.5 (−0.7, −0.4)	0.0 (0.0, 0.0)	−0.9 (−0.9, −0.8)	0.1 (0.1, 0.1)	−0.8 (−0.9, −0.7)
Sodium (mg)	2747 (2671, 2823)	4.8 (3.1, 6.5)	−84 (−117, −50)	0.5 (−1.8, 2.9)	−210 (−225, −195)	9.6 (7.6, 11.7)	−151 (−168, −134)

^1^Adjusted for season and holidays (spring [ref], summer, fall, holidays [week of Thanksgiving to week of New Year’s], winter).

^2^Baseline trend (per 4-week period from April 2015 to April 2017).

^3^Level change after franchise labeling in April 2017.

^4^Trend change (per 4-week period) after franchise labeling in April 2017.

^5^Level change after nationwide labeling in May 2018.

^6^Trend change (per 4-week period) after nationwide labeling in May 2018.

^7^To estimate the overall association at the end of the study, we calculated the predicted counterfactual value in the last week (i.e., a model that included only the baseline level, baseline trend, and seasonal covariates), subtracted this from the predicted actual value in the last week (i.e., a model that included the baseline level, baseline trend, all level and trend changes, and seasonal covariates), and calculated 95% CIs from 1,000 bootstrapped samples.

CI, confidence interval.

For other nutrients, we overall observed small level changes and minimal trend changes after each labeling period (Table 2). At the end of the study, the model-based estimates of nutrient content were lower for all nutrients than expected had labeling not been implemented (total fat: −3.0%; carbohydrates: −5.0%; protein: −5.1%; saturated fat: −1.8%; sugar: −7.0%; fiber: −4.4%; sodium: −5.0%). For some nutrients, these decreases offset the overall positive trends observed in the baseline period. For example, when comparing predicted nutrient content at the beginning and end of the study, we found slight improvements for saturated fat (12.6% of calories [April 2015] versus 12.0% of calories [April 2019]) and fiber (10.9 g/1,000 calories versus 11.6 g/1,000 calories). However, for other nutrients, the decrease at the end of the study did not offset positive baseline trends. For example, we did not find improvements for sugar (18.0% of calories [April 2015] versus 18.3% of calories [April 2019]) or sodium (2,748 mg versus 2,850 mg) at the end of the study.

When we ran models that assumed an intervention only at the time of franchise labeling (ignoring nationwide implementation), level and trend changes were different than in the main analyses (**S2 Table**). However, these models suggested end-of-study decreases in calorie and nutrient purchases that were very similar to results from our main analyses (e.g., 74-calorie/transaction decrease [95% CI: 67, 82]) for all nutrients except sodium (larger change of −208 mg/transaction, 95% CI: −224, −190).

We observed a stronger trend increase in calories/transaction after franchise labeling in lower-income census tracts than in higher-income census tracts (**Table 3**). By the end of the study, transactions in higher-income census tracts had on average 95 fewer calories (95% CI: 85, 107) than expected had labeling not occurred, but those in lower-income tracts had on average only 49 fewer calories (95% CI: 39, 59) than expected. This pattern was consistent across all nutrients.

**Table 3:** Interrupted time series for change in mean calories and nutrients purchased per transaction after restaurant calorie labeling implementation (April 2017) and after nationwide calorie labeling implementation (May 2018) by median income of restaurant census tracts^1^.

	β (95% CI)^2^						
Nutrient	Baseline level	Baseline trend^3^	Franchise level change^4^	Franchise trend change^5^	Nationwide level change^6^	Nationwide trend change^7^	Estimated change at end of study^8^
**Calories**							
Lower income	1,429 (1,391, 1,466)	1.8 (1.2, 2.4)	−48 (−62, −34)	4.0 (3.0, 5.0)	−82 (−91, −73)	−2.2 (−3.4, −1.1)	−49 (−59, −39)
Higher income	1,457 (1,407, 1,507)	2.4 (1.3, 3.5)	−60 (−81, −40)	2.6 (1.4, 3.9)	−82 (−90, −73)	−2.0 (−3.1, −0.8)	−95 (−107, −85)
**Fat (g)**							
Lower income	61.4 (59.6, 63.2)	0.0 (0.0, 0.0)	−0.6 (−1.4, 0.1)	0.2 (0.1, 0.2)	−2.5 (−3.0, −2.0)	−0.1 (−0.2, −0.1)	−0.9 (−1.4, −0.4)
Higher income	63.2 (60.6, 65.8)	0.0 (0.0, 0.1)	−1.8 (−3.0, −0.5)	0.1 (0.0, 0.2)	−2.4 (−2.8, −2.0)	−0.1 (−0.2, −0.1)	−2.9 (−3.5, −2.3)
**Carbohydrates (g)**							
Lower income	174.9 (170.7, 179.2)	0.4 (0.3, 0.5)	−9.8 (−11.2, −8.4)	0.6 (0.5, 0.7)	−9.3 (−10.3, −8.2)	−0.2 (−0.4, −0.1)	−6.6 (−8.0, −5.4)
Higher income	177.0 (171.7, 182.3)	0.5 (0.4, 0.6)	−10.5 (−12.3, −8.6)	0.3 (0.2, 0.5)	−10.1 (−11.2, −9.1)	−0.1 (−0.3, 0.0)	−13.1 (−14.3, −11.8)
**Protein (g)**							
Lower income	47.9 (46.3, 49.5)	0.0 (0.0, 0.1)	0.0 (−0.6, 0.7)	0.0 (−0.1, 0.0)	−2.7 (−3.0, −2.4)	0.1 (0.1, 0.1)	−2.3 (−2.6, −1.9)
Higher income	49.2 (47.1, 51.3)	0.0 (0.0, 0.1)	−0.2 (−1.0, 0.7)	−0.1 (−0.1, 0.0)	−2.4 (−2.7, −2.0)	0.1 (0.0, 0.1)	−3.0 (−3.4, −2.6)
**Saturated fat (g)**							
Lower income	19.9 (19.2, 20.5)	0.0 (0.0, 0.0)	−0.2 (−0.5, 0.1)	0.1 (0.0, 0.1)	−1.5 (−1.7, −1.4)	0.0 (0.0, 0.0)	0.0 (−0.2, 0.1)
Higher income	20.6 (19.7, 21.4)	0.0 (0.0, 0.0)	−0.6 (−1.2, −0.1)	0.1 (0.0, 0.1)	−1.4 (−1.5, −1.2)	0.0 (−0.1, 0.0)	−0.7 (−0.9, −0.5)
**Sugar (g)**							
Lower income	65.7 (63.5, 68.0)	0.1 (0.1, 0.2)	−1.7 (−2.5, −0.9)	0.0 (−0.1, 0.1)	−0.5 (−1.0, 0.0)	−0.1 (−0.2, 0.0)	−3.6 (−4.2, −2.9)
Higher income	64.4 (62.5, 66.2)	0.2 (0.1, 0.2)	−2.0 (−2.7, −1.4)	−0.1 (−0.1, 0.0)	−1.6 (−2.1, −1.1)	−0.1 (−0.1, 0.0)	−6.6 (−7.1, −6.0)
**Fiber (g)**							
Lower income	15.6 (15.2, 16.0)	0.0 (0.0, 0.0)	−0.6 (−0.7, −0.4)	0.0 (0.0, 0.0)	−0.9 (−0.9, −0.8)	0.1 (0.0, 0.1)	−0.6 (−0.7, −0.4)
Higher income	15.9 (15.4, 16.4)	0.0 (0.0, 0.1)	−0.5 (−0.7, −0.3)	0.0 (0.0, 0.0)	−0.9 (−1.0, −0.8)	0.1 (0.0, 0.1)	−1.0 (−1.2, −0.9)
**Sodium (mg)**							
Lower income	2,698 (2,610, 2,787)	4.5 (3.0, 5.9)	−75 (−121, −30)	1.6 (−0.5, 3.7)	−221 (−245, −196)	10.2 (8.0, 12.3)	−117 (−140, −95)
Higher income	2,796 (2,671, 2,920)	5.2 (2.2, 8.1)	−92 (−142, −41)	−0.5 (−4.7, 3.7)	−199 (−217, −182)	9.1 (5.6, 12.7)	−184 (−211, −156)

^1^Lower-income census tracts had a median income <$50,329, and higher-income census tracts had a median income >$50,329. In the US, a census tract is a small geographic unit (typically having a population size between 1,200 and 8,000 people) for which population-level demographic data are available [23].

^2^Adjusted for season and holidays (spring [ref], summer, fall, holidays [week of Thanksgiving to week of New Year’s], winter).

^3^Baseline trend (per 4-week period from April 2015 to April 2017).

^4^Level change after franchise labeling in April 2017.

^5^Trend change (per 4-week period) after franchise labeling in April 2017.

^6^Level change after nationwide labeling in May 2018.

^7^Trend change (per 4-week period) after nationwide labeling in May 2018.

^8^To estimate the overall association at the end of the study, we calculated the predicted counterfactual value in the last week (i.e., a model that included only the baseline level, baseline trend, and seasonal covariates), subtracted this from the predicted actual value in the last week (i.e., a model that included the baseline level, baseline trend, all level and trend changes, and seasonal covariates), and calculated 95% CIs from 1,000 bootstrapped samples.

CI, confidence interval.

We observed minimal reformulation of calories and nutrient content of the 100 top-selling items that remained on the menu in the pre- and post-labeling periods (**S3 Table**). Items that were newly introduced in 2018 did not have lower nutrient content than those introduced in 2016 to 2017 (i.e., pre-labeling). We also did not find statistically significant lower nutrient content of items newly introduced in 2019 (versus 2016 to 2017), though the point estimates were much stronger. For example, items newly introduced in 2019 had on average 108.5 fewer calories (95% CI: −16.9, 233.8, *p* = 0.09) than those newly introduced in 2016 to 2017. We also found that the median calorie content of all items offered at the restaurants decreased over time (from 290 calories in 2015 to 235 calories in 2019); nutrient content did not appear to change appreciably over time (**S4 Table**). Lastly, when exploring mechanisms for changing calories over time, we observed slight level increases in calories purchased per item after each labeling period, but these were followed by trend decreases that largely attenuated these associations (**S5 Table**). We observed small decreases in mean items/transaction after franchise labeling (β_franchise_ = −0.3 items/transaction, 95% CI: −0.3, −0.2, *p* < 0.0001) and after nationwide labeling (β_nation_ = −0.4 items/transaction, 95% CI: −0.5, −0.4, *p* < 0.0001), neither of which was followed by a trend change.

## Discussion

Data from this large natural experiment support an association of calorie labeling with small decreases in calorie and nutrient content of purchased fast food meals 2 years after initial franchise implementation and 1 year after nationwide implementation. By the end of the study, the average transaction contained an estimated 73 fewer calories (−4.7%) than expected had labeling not occurred. Labeling was also associated with overall small decreases in nutrient content of meals purchased that were consistent with calorie results. These results support modest improvements in purchased meal quality in response to calorie menu labeling.

Our previous analysis, which examined only franchise labeling over 1 year [14], found a similarly small level decrease and post-implementation trend increase in calories/transaction after labeling (small differences were apparent because the current study included 1 additional month of data after franchise labeling but before nationwide labeling). The present analysis indicates longer-term reductions in calories/transaction following nationwide implementation, albeit with an estimated increased trend of 1.2 calories/transaction per 4-week period. The reason for this increasing trend is unknown, but it may be explained if some customers stopped responding to the labels over time. Alternatively, the franchise could have engaged in other business practices to counteract labeling (e.g., marketing), though we could not examine this with our data. If the association persists over time, even the small reduction we observed could have an important population-level impact on energy balance and health [14,28].

The small decreases we observed in meals’ nutrient content (i.e., besides calories) are likely due to the fact that customers purchased overall less food after labeling. Because most of the nutrients we analyzed contribute to calories, reduced purchases of food overall would lead to reductions in nutrient content that were similar to the reduction in calories, which is what we observed (i.e., 3% to 5% decreases for calories, total fat, carbohydrates, and protein). Additionally, our exploratory analysis found that labeling was associated with decreases in purchased items/transaction but not with decreases in purchased calories/item. This finding is consistent with a previous study of labeling in a coffee shop chain, which found a 6% decrease in calories purchased post-labeling that was mostly driven by customers buying fewer items [15].

Although labeling was associated with reduced absolute nutrient content, the nutritional composition of meals (i.e., nutrient content relative to calorie content), which is an independent predictor of chronic disease risk [29], did not consistently change for the better. For example, at the end of the study, the predicted sugar content (18.3% of calories) and sodium content (2,850 mg) were slightly higher than at the beginning of the study. Even nutrients that did improve by the end of the study (e.g., saturated fat: 12.0%; fiber: 11.6*g*/1,000 calories) still had overall lower nutritional quality than US dietary recommendations. The 2020–2025 Dietary Guidelines for Americans recommends that adults consume <10% calories from saturated fat, about 14 g fiber/1,000 calories, and <2,300 mg sodium per day for reducing chronic disease risk [30]. It also recommends that adults consume <10% calories from added sugar, though we only had data on total sugar. Although we do not know whether purchased meals exceeded these limits for individuals (due to lack of individual-level data), the nutrient composition of meals suggests overall low quality, even after labeling.

We observed little evidence of menu reformulation among the top-selling items after labeling and found that the distribution of nutrient content for menu items was similar over time, suggesting an overall small role of changes in the nutrient content of restaurant offerings. It has been hypothesized that menu labeling would encourage restaurants to reduce the calorie content of their menu offerings, which would lower the calorie content of purchased meals without requiring changes to customer behavior. Our results that find minimal changes in the calorie content of continuously offered items are consistent with findings from other studies that have assessed post-labeling menu reformulation [17,18]. This has not yet been formally analyzed across all major restaurant chains in the US since nationwide implementation. At the same time, we observed that items newly introduced in 2019 had on average 109 fewer calories than items newly introduced in 2016 or 2017, though the 95% CI was wide and crossed the null due to small sample size. It is possible that labeling did not cause restaurants to reformulate their standard offerings but did cause them to introduce more lower-calorie items, which could improve customer meal choice. This should be analyzed in the future in a larger sample of restaurant chains.

Our stratified results by median household income of restaurant census tracts revealed a potential disparity in the effect of calorie labeling on customer purchase quality. By the end of the study period, the post-labeling decrease in calorie content in higher-income neighborhoods (−95 calories/transaction) was nearly twice that of the decrease in lower-income neighborhoods (−49 calories/transaction). Given that low socioeconomic status is associated with poorer diet quality and increased risk of obesity in the US and other high-income countries [31–33], this disparity could potentially worsen existing inequities in diet quality and nutrition-related disease. Because we did not have individual-level data, we cannot confirm whether there is a true disparity between individuals of different socioeconomic status. However, previous studies of individuals have found that those with higher incomes are more likely to see and use calorie menu labels than those with lower incomes [34–36]. Based on prior studies, other labeling systems (e.g., traffic light labels and warning labels) may be more effective than nutrient disclosures [37,38], particularly among low- and middle-income populations [39,40].

Most previous quasi-experimental studies in real-world settings in the US have not detected associations between calorie labeling and calorie content of meal purchases [7–12]. However, most of these studies were not powered to detect very small differences in calories purchased, took place in different areas of the US, and examined purchases over shorter periods of time. One previous study of similar size that used data from a large coffee shop chain found a 6% decrease in calories purchased after New York City’s 2008 implementation of calorie labeling, compared to cities that did not require labeling then [15]. Previous studies that examined changes in nutrient content have also been too small to detect the minor changes we observed [9,10,13]. Studies evaluating the effect of labeling on food purchases outside of the US are more limited, perhaps because other countries have implemented labeling more recently or done so on a voluntary basis [5]. Results of studies in Canada and Australia suggest improved customer meal choice in response to labeling [41–43]; these have either assessed many types of menu labeling interventions (i.e., not just calorie information) or have been conducted in laboratory settings, where results may not generalize to real-world settings.

This study has limitations. First, we did not have a control group, which limited our ability to adjust for time-varying confounders [44]; however, we adjusted for some population-level confounders (e.g., Hurricane Harvey). Moreover, ITS analyses are generally robust to individual-level confounders assuming the population remains stable over time [26]. Second, we did not know how many people were included as a part of each transaction. Because many transactions probably included meals for >1 person, the associations we observed are likely to be smaller for individuals. Third, if, labeling did not change the meal purchased but rather some post-purchasing behavior (e.g., if some people ate less of the meal), we might have underestimated the effect of labeling on individuals’ health. Fourth, given that all restaurants were located in southern states in the US, our results may not generalize to other parts of the US or to other countries. Fifth, like all statistical models, our models may have been misspecified (e.g., not adjusting for seasonality correctly), which could lead to bias. However, most sensitivity analyses that tested our modeling assumptions yielded similar results with the overall same conclusion. The sensitivity analyses that assumed level and trend changes only at the time of franchise labeling found different level and trend changes, but the predicted change at the end of the study was very similar to our main analysis. This implies robustness of our main finding that labeling was associated with decreased nutrient content of meals 2 years after franchise labeling. Another sensitivity analysis that used just 1 year of pre-implementation data suggested weaker associations after franchise labeling level than our main analysis. Although it is difficult to know which model better predicts the baseline trend, we preferred our main model because the additional data points could provide more stable estimation of the baseline trend. Our 2-year baseline main analysis also may have adjusted for seasonality better than the 1-year baseline analysis because it covered pre-labeling data from 2 of each season, instead of 1 each in the sensitivity analysis.

To the best of our knowledge, this study is the first to examine calorie labeling and restaurant purchases after nationwide implementation in the US. Among 104 restaurants, calorie labeling was associated with a small decrease in mean calories/transaction 2 years after initial franchise labeling and nearly 1 year after implementation of labeling nationwide. The absolute nutrient content of purchased meals decreased in a consistent way; however, the nutritional composition of meals was similar to pre-labeling levels and exceeded dietary recommendations. Further research should examine this policy in chain full-service restaurants and other food establishments where labels are required on prepared foods (e.g., supermarkets). In fast food settings, additional interventions and policies are likely necessary to further reduce the calorie content and improve the nutritional quality of meals.

## Supporting information

S1 Checklist STROBE checklist. (DOCX)

S1 Appendix Pre-analysis plan. (PDF)

S2 Appendix Supplemental methods. (DOCX)

S1 Table Interrupted time series (β [95% CI]) for change in mean calories purchased per transaction after restaurant calorie labeling implementation (April 2017) and after nationwide calorie labeling implementation (May 2018) for main and sensitivity analyses. (DOCX)

S2 Table Interrupted time series (β [95% CI]) for change in mean calories and mean nutrients purchased per transaction after franchise calorie labeling implementation, excluding model terms for nationwide labeling. (DOCX)

S3 Table Estimated change in mean nutrient values per item among continuously available items and newly introduced items. (DOCX)

S4 Table Median (IQR) and range of nutrients offered on restaurant menus in each year of the study. (DOCX)

S5 Table Interrupted time series (β [95% CI]) for level and trend change in mean calories purchased per transaction after franchise calorie labeling implementation (April 2017) and after nationwide calorie labeling implementation (May 2018). (DOCX)

## Abbreviations

ACA: Affordable Care Act
ACS: American Community Survey
CI: confidence interval
DOHMH: Department of Health and Mental Hygiene
ITS: interrupted time series
