# Supplementary material for: Changes in the calorie and nutrient content of purchased fast food meals after calorie menu labeling: A natural experiment
Source: PLoS Med. 2021 Jul 12;18(7):e1003714. doi: 10.1371/journal.pmed.1003714 (PMC8312920; doi:10.1371/journal.pmed.1003714)
Supplement: S1 Appendix — (PDF) [file pmed.1003714.s002.pdf]

Calorie labeling in fast-food restaurants (#19302)

Author(s)
Joshua Petimar (Harvard T.H. Chan School of Public Health) - jsp778@mail.harvard.edu
Jason Block (Harvard Medical School/Harvard Pilgrim Health Care Institute) - JBLOCK1@partners.org
Fang Zhang (Harvard Medical School/Harvard Pilgrim Health Care Institute) - fang_zhang@harvardpilgrim.org

Created: 02/05/2019 06:29 AM (PT)
Public: 03/28/2019 09:27 AM (PT)

1) Have any data been collected for this study already?
It's complicated. We have already collected some data but explain in Question 8 why readers may consider this a valid pre-registration nevertheless.

2) What's the main question being asked or hypothesis being tested in this study?
We hypothesize that implementation of menu calorie labeling at restaurants of a large fast-food chain will be associated with a modest decrease in mean calories purchased per transaction.

3) Describe the key dependent variable(s) specifying how they will be measured.
The dependent variable is mean calories purchased per transaction, which will be calculated using restaurant transaction/sales data provided by a large franchisee of an international fast-food company from March 2015 until April 2018. The data include a record of all items purchased on a weekly basis in each of 140 individual restaurants that are part of the franchisee's company, as well as the number of transactions made in each restaurant per week. We will assign all purchased items nutritional information primarily using Menustat, a database that contains nutrient data for foods and beverages offered in major restaurant chains in the U.S. (updated on a yearly basis). If this information is not available in Menustat, we will link purchased items to nutrition information from restaurant websites when available. We will sum the number of calories purchased in each restaurant per week and divide by the number of transactions to obtain mean calories purchased per week.

4) How many and which conditions will participants be assigned to?
The chain began labeling its menus with calorie information in April 2017. We will investigate the mean calories purchased per transaction before and after labeling went into effect.

5) Specify exactly which analyses you will conduct to examine the main question/hypothesis.
We will use an interrupted time series (ITS) to investigate whether there was a change in mean calories purchased per transaction after calorie labeling went into effect. Before conducting the ITS analysis, we will model the pre-intervention trend in the outcome using the two years of data before labeling implementation. We will consider potential time-varying covariates (e.g. season, meteorological events, socioeconomic measures of restaurant ZIP codes, etc.) to include in this model. We will additionally inspect the pre-intervention time series for linearity and consider adding polynomial terms or splines if the data exhibit nonlinear patterns.

After fitting the final pre-intervention model, we will conduct the ITS analysis by fitting a generalized linear mixed regression model with terms for week, intervention, an interaction term between week and intervention, and potential time-varying covariates. Restaurant location will be included as a random effect. This model allows for both a level change and slope change in the outcome following the intervention, which we believe are possible given the nature of the intervention and outcome. We also expect that there was a gradual rollout of calorie labeling on restaurant menu boards. We will account for a potential transition period by excluding two weeks of data both before and after the expected date of calorie labeling. We will conduct sensitivity analyses to test the robustness of our final model for calories purchased per transaction. These include modeling the pre-intervention trend using one year of data before calorie labeling instead of two years, restricting the analysis to restaurant locations that were available every week of the study, and excluding items that were imperfectly matched to nutrition information in Menustat.

6) Describe exactly how outliers will be defined and handled, and your precise rule(s) for excluding observations.
We will exclude restaurant locations with very low sales for a given week (<100 items sold); these are most likely data entry errors. We will additionally exclude purchases for which nutrition data could not be obtained in Menustat or on restaurant websites.

7) How many observations will be collected or what will determine sample size? No need to justify decision, but be precise about exactly how the number will be determined.
This analysis will include approximately 150 weeks of data, with an average of 100 restaurants available per week for a total of approximately 15,000 data points.

8) Anything else you would like to pre-register? (e.g., secondary analyses, variables collected for exploratory purposes, unusual analyses planned?)
We are not conducting any primary data collection in this study - the sales data are provided directly by the franchisee, while the nutrition data come from Menustat, a publicly available database. We therefore have no control or influence over the data collection process. Although some of the data are in our possession (i.e. most of the sales and nutrition data from 2015-2018 are available to us), we have not conducted any analyses of the data related to our research question as of submission of this pre-registration.

Available at https://aspredicted.org/4xx8v.pdf
(Permanently archived at http://web.archive.org/web/*/https://aspredicted.org/4xx8v.pdf)

In April 2017, around the same time that calorie labeling began, the franchise changed methods of recording sales data such that both combination meals and the individual items that made up the combo were recorded (with the exception of beverages). Conversely, before April 2017, only combo meals were recorded, but not their constituents. As a result, items purchased in combos were double-counted after April 2017. To correct this, we identified all combos purchased before April 2017 and matched them to individual items that came with the combo using restaurant website information. We then deleted the combo purchases before and after April 2017, resulting in a standardized/corrected number of items purchased in both periods. One challenge of this approach is that any changes to the default combo (e.g. substituting beef for chicken in an entrée) were recorded after April 2017 but not before April 2017, leading to potential differential measurement error. To test the robustness of our results, which will primarily assume the default option for combos purchased before April 2017, we will rerun our final models where we consider all possible options for items that come with combos and will use a weighted average of these possible options from a la carte purchases (weighted on a weekly basis). We will additionally conduct analyses in which we separately assume that the option with the maximum and minimum number of calories were chosen, which will give us a range of the highest and lowest number of calories possibly purchased in the period before April 2017. We will explore potential effect modification of the association between calorie labeling and calories purchased per transaction by demographic characteristics of ZIP codes of restaurants (i.e. median household income and percent non-white residents).

In secondary analyses, we will rerun our final model for specific nutrients of interest to examine any changes in the nutrient composition of items purchased after calorie labeling.

Available at https://aspredicted.org/4xx8v.pdf

(Permanently archived at http://web.archive.org/web/*/https://aspredicted.org/4xx8v.pdf)
