# Supplementary material for: Changes in the calorie and nutrient content of purchased fast food meals after calorie menu labeling: A natural experiment
Source: PLoS Med. 2021 Jul 12;18(7):e1003714. doi: 10.1371/journal.pmed.1003714 (PMC8312920; doi:10.1371/journal.pmed.1003714)
Supplement: S2 Appendix — (DOCX) [file pmed.1003714.s003.docx]

**S2 Appendix**

**1. Changes in analysis from pre-registered analysis plan**

Our analysis deviated slightly from what was outlined in our pre-registered analysis plan, including both changes made to the analysis and additional analyses that were not planned ahead of time:

Changes

- We did not include random slopes in our models because the models did not converge with these additional random effects. We therefore only included random intercepts.
- We did not conduct sensitivity analyses excluding items that were not perfectly matched to nutrition information in Menustat. Our first paper using these data (Petimar et al. 2019, BMJ) found that only 3% of sold items did not have identical matches to Menustat nutrition information, and results were similar after we excluded these items. We therefore did not repeat this analysis here.
- We excluded restaurant-weeks with <1000 items sold in a given week (instead of <100 as proposed) because there was one additional restaurant-week with >100 items sold but still appeared to be an outlier upon visual inspection.
- We did not conduct sensitivity analyses that imputed combo items in proportion to the amount sold a la carte. Our previously published paper explored this (Petimar et al. 2019, BMJ) and found very similar results when doing this imputation. Because this issue only affected the pre-labeling data, we believe that our current study, which extends the post-labeling data, will not show appreciably different results.

Additions

- We conducted sensitivity analyses aggregating data into 4-week periods of time instead of 1-week periods because we were concerned about week-to-week variability affecting our estimates, though the results of this analysis were very similar to our main results.
- We conducted sensitivity analyses including sine and cosine terms for season to see if this provided better adjusted for seasonality.
- We ran a model not including terms for nationwide implementation (not proposed in our analysis plan because our first paper did not extend into this period).
- We explored possible menu reformulation and changes in nutrient content of menu items to potentially identify the role of reformulation in the associations we observed.
- We ran models examining calories/item and items/transaction to potentially identify mechanisms that could explain the associations we observed.

**2. Obtaining nutrition information for purchased items**

The New York City Department of Health and Mental Hygiene did not release a Menustat database in 2019. We therefore created our own database with nutrition information of items offered at the top revenue-generating restaurants in 2019 (with guidance from the NYC DOHMH). This data capture was completed in January 2019 to be consistent with previous years of Menustat.

For each restaurant listed in 2018 Menustat, we took screenshots of menu information (i.e. item names and descriptions), photos, and all available nutrition information from the restaurant’s U.S. website. We also downloaded PDFs of menus, if available, for each restaurant. For items that were customizable, we captured nutrition information for the default build only. If available, we also collected information on items’ food category, serving size, and other descriptive variables (e.g. kids meals or regional offerings). We entered all items into a dataset and assigned them the same Menustat ID as in previous years if the item was offered previously. We did this by matching items on keywords in the item name or description. New items were assigned a unique Menustat ID. Initial data entry was completed by research assistants and double-checked by other members of the research staff.

**3. Statistical model**

Calories/transaction = β_0_ + β_1_week + β_2_franchise_label + β_3_week_franchise_label + β_4_nation_label + β_5_week_nation_label + β_6_summer + β_7_fall + β_8_winter + β_9_holidays + b_0i_ + b_1i_Z_1it_ + b_2i_ Z_2it_ + e_it_

where i indexes location, t indexes weeks of measurement

β_0_ = baseline mean calories/transaction

β_1_ = baseline weekly trend in calories/transaction

β_2_ = level change in calories/transaction after franchise labeling

β_3_ = trend change in calories/transaction after franchise labeling

β_4_ = level change in calories/transaction after nationwide labeling

β_5_ = trend change in calories/transaction after nationwide labeling

β_6_ = change in calories/transaction during the summer

β_7_ = change in calories/transaction during the fall

β_8_ = change in calories/transaction during the winter

β_9_ = change in calories/transaction during the holidays

b_0i_ = random intercept for location i

b_1i_ = random intercept of franchise label period for location i

b_2i_ = random intercept of nationwide label period for location i

Z_1it_= week of the observation

Z_2it_ = franchise label of the observation

Z_3it_ = nationwide label of the observation

e_it_= measurement error for location i at time t

This model assumes that b_0i,_ b_1i,_ b_2i,_ and e_it_ are all independently normally distributed random variables with mean 0 and some variance
