# Supplementary material for: Changes in the calorie and nutrient content of purchased fast food meals after calorie menu labeling: A natural experiment
Source: PLoS Med. 2021 Jul 12;18(7):e1003714. doi: 10.1371/journal.pmed.1003714 (PMC8312920; doi:10.1371/journal.pmed.1003714)
Supplement: S1 Table — (DOCX) [file pmed.1003714.s004.docx]

| **S1 Table.** **Interrupted time series for change in mean calories purchased per transaction after restaurant calorie labeling implementation (April 2017) and after nationwide calorie labeling implementation (May 2018) for main and sensitivity analyses** | | | | | | | | |
| --- | --- | --- | --- | --- | --- | --- | --- | --- |
|  | | **β (95% CI)^1^** | | | | | | |
| Analysis | | Baseline level | Baseline trend^2^ | Franchise level change^3^ | Franchise trend change^4^ | Nationwide level change^5^ | Nationwide trend change^6^ | Estimated change at end of study^7^ |
| **Main analysis** | | 1443 (1411, 1474) | 2.1 (1.5, 2.7) | -54 (-67, -42) | 3.3 (2.5, 4.1) | -82 (-88, -76) | -2.1 (-2.9, -1.3) | -73 (-81, -65) |
| **Sensitivity analysis** | |  |  |  |  |  |  |  |
|  | One year of pre-labeling data | 1496 (1468, 1524) | -1.8 (-2.7, -0.8) | -27 (-42, -13) | 5.8 (4.7, 6.9) | -64 (-70, -59) | -1.9 (-2.7, -1.1) | 43 (22, 63) |
|  | Restaurants available every week | 1446 (1411, 1480) | 2.3 (1.9, 2.7) | -36 (-46, -26) | 3.7 (2.6, 4.7) | -81 (-90, -73) | -2.0 (-3.3, -0.7) | -70 (-80, -61) |
|  | Average of 4 weeks as unit of analysis (instead of 1-week periods) | 1447 (1416, 1479) | 2.1 (1.5, 2.7) | -55 (-66, -43) | 3.3 (2.5, 4.2) | -76 (-82, -71) | -2.5 (-3.3, -1.6) | -71 (-80, -65) |
|  | Including sine and cosine terms for season^8^ | 1434 (1403, 1465) | 2.6 (2.0, 3.2) | -73 (-85, -61) | 4.5 (3.6, 5.3) | -100 (-107, -94) | -2.6 (-3.4, -1.8) | -85 (-86, -85) |
| ^1^Adjusted for season and holidays (spring [ref], summer, fall, holidays [week of Thanksgiving to week of New Year's], winter) | | | | | | | | |
| ^2^Baseline trend (per 4-week period from April 2015 to April 2017) | | | | | | | | |
| ^3^Level change after franchise labeling in April 2017 | | | | | | | | |
| ^4^Trend change (per 4-week period) after franchise labeling in April 2017 | | | | | | | | |
| ^5^Level change after nationwide labeling in May 2018 | | | | | | | | |
| ^6^Trend change (per 4-week period) after nationwide labeling in May 2018 | | | | | | | | |
| ^7^To estimate the overall association at the end of the study, we calculated the predicted counterfactual value in the last week (i.e., a model that included only the baseline level, baseline trend, and seasonal covariates), subtracted this from the predicted actual value in the last week (i.e., a model that included the baseline level, baseline trend, all level and trend changes, and seasonal covariates), and calculated 95% CIs from 1000 bootstrapped samples. | | | | | | | | |
| ^8^This model did not include indicators for season; instead, we included sine and cosine terms for season assuming a period of one year (i.e. sin(2π*week of year/52) and cos(2π*week of year/52)) | | | | | | | | |
