# Supplementary material for: Changes in the calorie and nutrient content of purchased fast food meals after calorie menu labeling: A natural experiment
Source: PLoS Med. 2021 Jul 12;18(7):e1003714. doi: 10.1371/journal.pmed.1003714 (PMC8312920; doi:10.1371/journal.pmed.1003714)
Supplement: S2 Table — (DOCX) [file pmed.1003714.s005.docx]

| **S2 Table. Interrupted time series for change in mean calories and mean nutrients purchased per transaction after franchise calorie labeling implementation, excluding model terms for nationwide labeling** | | | | | | |
| --- | --- | --- | --- | --- | --- | --- |
|  | | **β (95% CI]^1^** | | | | |
| Nutrient | | Baseline level | Baseline trend^2^ | Franchise level change^3^ | Franchise trend change^4^ | Estimated change at end of study^5^ |
| **Calories per transaction** | | 1445 (1414, 1476) | 1.8 (1.1, 2.4) | -13 (-26, -1) | -2.2 (-2.9, -1.5) | -74 (-82, -67) |
| **Absolute nutrient content per transaction** | | |  |  |  |  |
|  | Fat (g) | 62.3 (60.8, 63.9) | 0.0 (0.0, 0.0) | 0.3 (-0.4, 1.0) | -0.1 (-0.1, 0.0) | -1.7 (-2.1, -1.3) |
|  | Carbohydrates (g) | 176.2 (172.8, 179.5) | 0.4 (0.3, 0.5) | -5.5 (-6.7, -4.4) | -0.2 (-0.2, -0.1) | -10.3 (-11.1, -9.4) |
|  | Protein (g) | 48.7 (47.4, 50.0) | 0.0 (0.0, 0.1) | 0.6 (0.1, 1.1) | -0.1 (-0.2, -0.1) | -3.2 (-3.5, -2.9) |
|  | Saturated fat (g) | 20.3 (19.7, 20.8) | 0.0 (0.0, 0.0) | 0.2 (0.0, 0.5) | 0.0 (0.0, 0.0) | -0.5 (-0.6, -0.3) |
|  | Sugar (g) | 65.0 (63.6, 66.5) | 0.1 (0.1, 0.2) | -1.1 (-1.6, -0.6) | -0.1 (-0.2, -0.1) | -4.9 (-5.3, -4.5) |
|  | Fiber (g) | 15.8 (15.5, 16.1) | 0.0 (0.0, 0.0) | -0.4 (-0.5, -0.3) | 0.0 (0.0, 0.0) | -1.1 (-1.2, -1.0) |
|  | Sodium (mg) | 2757.6 (2681.8, 2833.4) | 4.1 (2.4, 5.8) | -36.9 (-71.5, -2.3) | -6.2 (-8.2, -4.2) | -208 (-224, -190) |
| ^1^Adjusted for season and holidays (spring [ref], summer, fall, holidays [week of Thanksgiving to week of New Year's], winter) | | | | | | |
| ^2^Baseline trend (per 4-week period from April 2015 to April 2017) | | | | | | |
| ^3^Level change after franchise labeling in April 2017 | | | | | | |
| ^4^Trend change (per 4-week period) after franchise labeling in April 2017 | | | | | | |
| ^5^To estimate the overall association at the end of the study, we calculated the predicted counterfactual value in the last week (i.e., a model that included only the baseline level, baseline trend, and seasonal covariates), subtracted this from the predicted actual value in the last week (i.e., a model that included the baseline level, baseline trend, the franchise level and trend change, and seasonal covariates), and calculated 95% CIs from 1000 bootstrapped samples. | | | | | | |
