# Supplementary material for: Changes in the calorie and nutrient content of purchased fast food meals after calorie menu labeling: A natural experiment
Source: PLoS Med. 2021 Jul 12;18(7):e1003714. doi: 10.1371/journal.pmed.1003714 (PMC8312920; doi:10.1371/journal.pmed.1003714)
Supplement: S3 Table — (DOCX) [file pmed.1003714.s006.docx]

| **S3 Table. Estimated change in mean nutrient values per item among continuously available items and newly introduced items** | | | | |
| --- | --- | --- | --- | --- |
| Nutrient | | Pre period (2015-2017)^1^ | 2018 | 2019 |
| **Among top 100 high-selling items that were sold in each period^2^** | | | |  |
|  | Calories | 0.0 (ref) | -2.5 (-4.5, -0.5) | -3.2 (-5.3, -1.1) |
|  | Total fat (g) | 0.0 (ref) | -0.3 (-0.5, -0.1) | -0.3 (-0.5, -0.2) |
|  | Carbohydrates (g) | 0.0 (ref) | 0.0 (-0.3, 0.2) | 0.3 (0.0, 0.6) |
|  | Protein (g) | 0.0 (ref) | -0.2 (-0.3, -0.1) | -0.3 (-0.4, -0.2) |
|  | Saturated fat (g) | 0.0 (ref) | -0.1 (-0.2, 0.0) | -0.1 (-0.2, 0.0) |
|  | Sugar (g) | 0.0 (ref) | -0.1 (-0.3, 0.0) | -0.2 (-0.3, -0.1) |
|  | Fiber (g) | 0.0 (ref) | 0.0 (-0.1, 0.1) | 0.0 (0.0, 0.1) |
|  | Sodium (mg) | 0.0 (ref) | -4.5 (-11.3, 2.2) | -4.2 (-11.1, 2.8) |
| **Among top 100 high-selling items that were sold every week of the study^2,3^** | | | | |
|  | Calories | 0.0 (ref) | -2.6 (-5.0, -0.2) | -1.9 (-4.3, 0.5) |
|  | Total fat (g) | 0.0 (ref) | -0.2 (-0.4, 0.0) | -0.2 (-0.3, 0.0) |
|  | Carbohydrates (g) | 0.0 (ref) | 0.0 (-0.2, 0.3) | 0.3 (0.1, 0.6) |
|  | Protein (g) | 0.0 (ref) | -0.2 (-0.3, -0.1) | -0.1 (-0.3, 0.0) |
|  | Saturated fat (g) | 0.0 (ref) | -0.1 (-0.2, 0.0) | -0.1 (-0.2, 0.0) |
|  | Sugar (g) | 0.0 (ref) | -0.1 (-0.3, 0.0) | -0.2 (-0.3, 0.0) |
|  | Fiber (g) | 0.0 (ref) | 0.0 (0.0, 0.1) | 0.0 (0.0, 0.1) |
|  | Sodium (mg) | 0.0 (ref) | -3.1 (-9.8, 3.6) | 1.0 (-5.7, 7.7) |
| **Items newly introduced^4^** | | |  |  |
|  | Calories | 0.0 (ref) | 14.9 (-98.7, 128.5) | -108.5 (-233.8, 16.9) |
|  | Total fat (g) | 0.0 (ref) | -0.6 (-8.2, 7.0) | -6.1 (-14.4, 2.3) |
|  | Carbohydrates (g) | 0.0 (ref) | 6.9 (-8.6, 22.3) | -11.1 (-28.2, 6.0) |
|  | Protein (g) | 0.0 (ref) | -1.8 (-6.3, 2.7) | -3.2 (-8.1, 1.8) |
|  | Saturated fat (g) | 0.0 (ref) | -0.1 (-1.8, 1.9) | -1.4 (-3.4, 0.7) |
|  | Sugar (g) | 0.0 (ref) | 3.7 (-11.0, 18.3) | -3.7 (-20.0, 12.5) |
|  | Fiber (g) | 0.0 (ref) | 0.8 (-0.7, 2.4) | 0.5 (-1.3, 2.2) |
|  | Sodium (mg) | 0.0 (ref) | -66.9 (-298.0, 164.1) | -186.6 (-441.6, 68.4) |
| ^1^The reference period was 2016-2017 for analyses of newly introduced items | | | | |
| ^2^Estimates represent the mean within-item change in the nutrient of interest for the year of interest compared to the pre-labeling period | | | | |
| ^3^Same as above analysis but restricted to items that were sold in each week of the study | | | | |
| ^4^This analysis is restricted to items that were not sold in 2015 but were sold at some point after. The estimates represent the mean change in nutrient content for items that were newly introduced in 2018 (n=25) or 2019 (n=19) compared to those that were newly introduced before labeling (n=56). | | | | |
