# Supplementary material for: Changes in the calorie and nutrient content of purchased fast food meals after calorie menu labeling: A natural experiment
Source: PLoS Med. 2021 Jul 12;18(7):e1003714. doi: 10.1371/journal.pmed.1003714 (PMC8312920; doi:10.1371/journal.pmed.1003714)
Supplement: S4 Table — (DOCX) [file pmed.1003714.s007.docx]

| **S4 Table. Median (IQR) and range of nutrients offered on restaurant menus in each year of the study** | | | | | | |
| --- | --- | --- | --- | --- | --- | --- |
| **Nutrient** | | **2015** | **2016** | **2017** | **2018** | **2019** |
| **N items** | | 274 | 279 | 277 | 263 | 234 |
| **Calories** | |  |  |  |  |  |
|  | Minimum | 0 | 0 | 0 | 0 | 0 |
|  | Median (IQR) | 290 (170, 450) | 280 (170, 440) | 270 (170, 440) | 260 (170, 420) | 235 (160, 410) |
|  | Maximum | 1160 | 1160 | 1120 | 1210 | 1120 |
| **Total fat (g)** | |  |  |  |  |  |
|  | Minimum | 0 | 0 | 0 | 0 | 0 |
|  | Median (IQR) | 11 (0, 20) | 11 (0, 19) | 10 (0, 19) | 11 (0, 19) | 11 (0, 18) |
|  | Maximum | 62 | 64 | 67 | 67 | 59 |
| **Carbohydrates (g)** | |  |  |  |  |  |
|  | Minimum | 0 | 0 | 0 | 0 | 0 |
|  | Median (IQR) | 39 (16, 60) | 37 (16, 58) | 37 (15, 58) | 38 (16, 55) | 34 (15, 54) |
|  | Maximum | 216 | 234 | 145 | 145 | 143 |
| **Protein (g)** | |  |  |  |  |  |
|  | Minimum | 0 | 0 | 0 | 0 | 0 |
|  | Median (IQR) | 8 (0, 17) | 9 (0, 17) | 8 (0, 17) | 8 (0, 16) | 8 (0, 16) |
|  | Maximum | 41 | 41 | 41 | 39 | 39 |
| **Saturated fat (g)** | |  |  |  |  |  |
|  | Minimum | 0 | 0 | 0 | 0 | 0 |
|  | Median (IQR) | 3 (0, 6) | 4 (0, 6) | 3 (0, 6) | 3 (0, 6) | 3 (0, 5) |
|  | Maximum | 37 | 37 | 37 | 37 | 25 |
| **Sugar (g)** | |  |  |  |  |  |
|  | Minimum | 0 | 0 | 0 | 0 | 0 |
|  | Median (IQR) | 4 (1, 15) | 3 (1, 11) | 3 (1, 15) | 3 (1, 22) | 3 (1, 10) |
|  | Maximum | 216 | 228 | 145 | 115 | 113 |
| **Dietary fiber (g)** | |  |  |  |  |  |
|  | Minimum | 0 | 0 | 0 | 0 | 0 |
|  | Median (IQR) | 2 (0, 4) | 2 (0, 4) | 2 (0, 4) | 1 (0, 4) | 2 (0, 4) |
|  | Maximum | 19 | 19 | 14 | 13 | 18 |
| **Sodium (mg)** | |  |  |  |  |  |
|  | Minimum | 0 | 0 | 0 | 0 | 0 |
|  | Median (IQR) | 490 (115, 960) | 490 (130, 980) | 460 (110, 980) | 435 (110, 975) | 460 (120, 910) |
|  | Maximum | 2930 | 2930 | 2930 | 3370 | 2930 |
