# Supplementary material for: Changes in the calorie and nutrient content of purchased fast food meals after calorie menu labeling: A natural experiment
Source: PLoS Med. 2021 Jul 12;18(7):e1003714. doi: 10.1371/journal.pmed.1003714 (PMC8312920; doi:10.1371/journal.pmed.1003714)
Supplement: S5 Table — (DOCX) [file pmed.1003714.s008.docx]

| **S5 Table. Interrupted time series for level and trend change in mean calories purchased per transaction after franchise calorie labeling implementation (April 2017) and after nationwide calorie labeling implementation (May 2018)** | | |
| --- | --- | --- |
| Analysis | | β (95% CI)^1^ |
| **Calories Per Item** | |  |
|  | Baseline kcal/item | 306 (303, 309) |
|  | Baseline trend (per 4-week period) in kcal/item | -0.1 (-0.2, -0.1) |
|  | Franchise level change in kcal/item | 4.3 (2.4, 6.1) |
|  | Franchise trend change (per 4-week period) in kcal/item | -0.6 (-0.7, -0.4) |
|  | Nationwide level change in kcal/item | 10.7 (9.9, 11.4) |
|  | Nationwide trend change (per 4-week period) in kcal/item | -1.1 (-1.2, -1.0) |
| **Items Per Transaction** | |  |
|  | Baseline items/transaction | 4.7 (4.6, 4.9) |
|  | Baseline trend (per 4-week period) in items/transaction | 0.0 (0.0, 0.0) |
|  | Franchise level change in items/transaction | -0.3 (-0.3, -0.2) |
|  | Franchise trend change (per 4-week period) in items/transaction | 0.0 (0.0, 0.0) |
|  | Nationwide level change in items/transaction | -0.4 (-0.5, -0.4) |
|  | Nationwide trend change (per 4-week period) in items/transaction | 0.0 (0.0, 0.0) |
| ^1^Adjusted for season and holidays (spring [ref], summer, fall, holidays [week of Thanksgiving to week of New Year's], winter) | | |
